# Principles of alternative gerontology

**DOI:** 10.18632/aging.100931

**Published:** 2016-03-25

**Authors:** Tomasz Bilinski, Aneta Bylak, Renata Zadrag-Tecza

**Affiliations:** ^1^ Department of Biochemistry and Cell Biology, University of Rzeszow, 35-601 Rzeszow, Poland; ^2^ Department of Environmental Biology, University of Rzeszow, 35-601 Rzeszow, Poland

**Keywords:** aging, longevity, senescence, energy, life program, growth, regeneration

## Abstract

Surveys of taxonomic groups of animals have shown that contrary to the opinion of most gerontologists aging is not a genuine trait. The process of aging is not universal and its mechanisms have not been widely conserved among species. All life forms are subject to extrinsic and intrinsic destructive forces. Destructive effects of stochastic events are visible only when allowed by the specific life program of an organism. Effective life programs of immortality and high longevity eliminate the impact of unavoidable damage. Organisms that are capable of agametic reproduction are biologically immortal. Mortality of an organism is clearly associated with terminal specialisation in sexual reproduction. The longevity phenotype that is not accompanied by symptoms of senescence has been observed in those groups of animals that continue to increase their body size after reaching sexual maturity. This is the result of enormous regeneration abilities of both of the above-mentioned groups. Senescence is observed when: (i) an organism by principle switches off the expression of existing growth and regeneration programs, as in the case of imago formation in insect development; (ii) particular programs of growth and regeneration of progenitors are irreversibly lost, either partially or in their entirety, in mammals and birds.

“We can't solve problems by using the same kind of thinking we used when we created them.” (Ascribed to Albert Einstein)

## Rationale

After the proclaimed emergence of the “new aging science” [1, 2] and the subsequent response by the representatives of “old aging science” [3], another approach, referred to as “alternative aging sciences” or “alternative gerontology”, was proposed, questioning the validity of basic premises of the two earlier approaches. The new approach rejects the underlying assumption of experimental gerontology that aging is a universal and genuine process and its mechanisms are conserved in all organisms. We propose this new and revolutionary perspective on gerontology based on the following reasoning. As presented earlier [4], life on Earth is a consequence of negentropy, which allows for creating various life structures using the external source of solar energy and the conserved information.

### All living things are subject to various adverse stochastic processes

Numerous extrinsic and intrinsic forces act on living things, constantly damaging the existing structures. Living organisms can either avoid some of these destructive forces or protect themselves by evolving appropriate mechanisms at the cellular and/or organismal level. Some damage, however, is unavoidable. The mechanisms that evolved in response to that problem may be broken down into several groups: (i) repair and replacement (turnover) mechanisms encountered at the cellular level and (ii) cell replacement and regeneration mechanisms at the organismal level. The forces described as destructive are diverse and depend on the environment and the organism [5], and therefore the actual damage is not necessarily the same in every case. Moreover, clearly damaged, useless or simply ballast molecules are formed as a consequence of natural imprecision of all biological processes or as a side effect of particular processes, such as formation of misfolded proteins, rDNA circles, or molecular sulphur granules observed in some photosynthesising bacteria. Hence, all life forms have to cope with various negative events. The cellular-level mechanisms are almost universal in the sense that mechanisms of solving most of the problems exist in cells of all living things. These protective and reparative mechanisms are encountered in all types of cells, and are similar in quantitative as well as qualitative terms. They are either maximally effective, or their intensity can be strongly and rapidly increased when necessary. This is a result of more than one giga-year of evolution of cellular systems. On the other hand, the organismal-level protective/repair/regeneration mechanisms evolved later, and are continuously modified under the pressure of constant environmental changes. These mechanisms have to be adapted to the chosen life strategies of a clade. As an example, the general structure of plants, invertebrates and vertebrates are fundamentally different, both in terms of their life strategies and in terms of presence or absence of various physiological processes. Consequently, the only universal phenomenon usually associated with aging processes is the unavoidability of adverse effects of extrinsic or intrinsic forces on an organism.

### Damage itself does not mean aging

It is generally believed that senescence, which alongside mortality is considered part of the aging process, is a consequence of operation of various adverse forces that are often associated with the effects of the Second Law of Thermodynamics on living things. Senescence, however, is observed only if the effects of these negative events have not been eliminated. When both the cellular- and organismal-level mechanisms are efficient, no effects of damage can be visible, at least in the long run. Effectiveness of these processes depends on external energy supply as well as the existence or availability of an appropriate program (information). Consequently, senescence means accumulation of various types of damage or ballast, both cellular and extracellular. In multicellular organisms, damage leading to senescence additionally means death of cells that are not replaced by new ones.

One can conclude, therefore, that senescence takes place only if allowed by low effectiveness of life programs of a particular organism. In other words, senescence is a result of allowing for manifestation of unavoidable effects of various adverse forces. As shown below, the degree of that allowance is different in various clades. Considering that the same forces can disrupt various organismal functions in varying ways depending on a particular organism, no universal mechanism of aging can exist. For example, oxidative damage to cells of *Saccharomyces cerevisiae* does not include various destructive processes resulting from peroxidation of polyunsaturated fatty acids, as the latter are not produced by the species [6]. On the other hand, accumulation of rDNA circles noted in yeast [7] is not found in human cells where an open mitosis process is observed.

### Aging is not a genuine trait

Aging evolved only as a side effect of the choice of a particular life strategy of a clade. As such, it corresponds perfectly to the term “spandrel” introduced by Gould and Lewontin [8]. With that in mind, gerontologists would be amiss to look for any universal mechanisms of aging because they simply do not exist. As a rational consequence, in order to explain the mechanisms of human aging, it is necessary to use the closest possible relatives of human beings as model organisms of gerontology. This opinion perfectly corresponds to the opinion of the representatives of “old aging sciences”[9]. Accepting the interpretation that the incidence and nature of aging processes are side effects of the chosen life strategies rather than genuine traits suggests the need to transform the methodological approach to the phenomenon.

## Basic definitions

Because of the semantic chaos in gerontology [10], we have to begin with defining the meaning of the term “aging” which will be used consistently throughout this paper. Aging is regarded as a progressive loss of function (including fertility) and increasing mortality with advancing age. Negative time-dependent changes are also structural. In other words, aging means appearance of various symptoms of senescence and unavoidable approach of death. A logical consequence of accepting this definition is that we cannot claim that unavoidability of death itself is necessarily a consequence of aging, if it is not accompanied by clear symptoms of senescence. One important point is that senescence has to be progressive rather than of an abrupt nature. For instance, premature death of the mayfly or Pacific salmon is not, in fact, preceded by senescence. Similarly, in the non-animal model of aging studies using the budding yeast, the rate of reproduction is very high throughout the majority of the organism's life, except in one or two generations (out of several tens) before reproductive cessation and eventual death. Knowing that the rate of reproduction requires perfectly coordinated synthesis of all elements of the cell, one can claim that death of yeast cells is not preceded by symptoms of senescence. The proximal cause of death is clearly a side effect of life programs (choice of budding mechanism of cytokinesis in yeast [11], failure of the developmental program of the digestive tract formation in the imago of the mayfly [12, 13] or hormonal/behavioural changes in the Pacific salmon which are absent in its close Atlantic cousin [14, 15]). Similarly, the European eel can live for 80 years in captivity, whereas if allowed to reproduce, it dies soon after spawning at a much younger age. These premature cases of death have, therefore, nothing to do with senescence or aging generally, and can be paralleled to cases of death of males of various species. They are clearly effects of life programs or mutations acquired by the species resulting in mortality after fulfilling the animal's reproductive functions.

It is hard to determine when human senescence starts. It seems reasonable to assume that it starts at the end of the adolescence period. During this period our organism reaches not only the sexual maturity, but also the final body size. In other words, aging starts when we reach the moment of adulthood. Initially, the effects of the processes leading to senescence are not easily measurable, yet the sum of these tiny and difficult-to-quantify changes allows us, for example, to estimate the age of another human being. Also, in insects undergoing complete transformation, we clearly distinguish the larval period during which the organism continuously grows retaining all cell replacement and regenerative capacities of the species. No organismal-level symptoms of senescence are therefore visible at this developmental step. During pre-imaginal (pupae) phase, all body structures are completely rebuilt and the imago emerges as a completely new entity mostly from a small structure named imaginal disk. Then senescence processes start immediately but their symptoms become visible later. Again, obvious senescence appears in sexually mature organisms who are no longer able to increase their size, but is not observed in animals that grow continuously. They behave as if “young forever”, corresponding to the continuously adolescent forms of mammals.

## Goals of gerontological studies

The problem of aging of animals practically does not exist in natural populations. Animals in the wild rarely survive until the symptoms of senescence become visible. As a biological science, gerontology is now strongly supported not so much for transcendental reasons, but rather because the age structure of the developed societies will soon create economic and social problems. The most important goal for such studies is to diminish the costs of population aging. Geriatrics needs a scientific basis for improving medical practice. Consequently, the aim of gerontology is to prevent the most life-devastating symptoms of senescence. Therefore, the basic role of gerontology, at least in the short term, should be identifying mechanisms that slow down and minimise the effects of senescence. In other words, the role of gerontology is not to extend the maximum lifespan above the limit characteristic for the given species. However, rather disappointingly, this is precisely what experimental gerontologists have been doing: rather than looking for mutants of various organisms in which the symptoms of senescence appear later or are less detrimental, they have been mainly looking for mutants with increased lifespan.

## Role of the Second Law of Thermodynamics in aging

The crucial role of the Second Law of Thermodynamics in aging has been one of the motifs of the discussion on the origin of aging for decades. Recently, a conference entitled “The second law of thermodynamics and the origins of biological aging” has been organised in 2014 by L. Hayflick and W. Bortz. The statement of purpose proclaims that: *“There are only two fundamental ways in which age changes can occur. They can arise either as the result of a purposeful program driven by genes or by random stochastic or accidental events”*. As the existence of the purposeful aging program has generally been rejected, the authors conclude that the Second Law of Thermodynamics plays a significant, if not exclusive, role in the events that lead to age-related changes at higher levels of complexity.

The chief fallacy of these statements is that they propose a false alternative. Between the purposeful program of aging and the stochastic events there are the earlier mentioned phenomena which Gould and Lewontin named “spandrels” [8]. The term stands for non-genuine traits that are by-products of the chosen original life programs that evolved and were shaped by forces of natural selection. In other words, spandrels are phenomena which in a natural but secondary way accompany other phenomena and did not evolve by themselves. Aging is a spandrel. In the following paragraphs, we are going to discuss cases where senescence and death result mainly from stochastic reasons and those which are determined by genetic (epigenetic) programs.

To begin, it is vital to make a clear distinction between the effects of the omnipresent adverse stochastic or accidental processes leading to wear and tear and the appearance of senescence. For example, insects, mammals and birds have developed three independent mechanisms of flight. In mammals all wings are built from live cells. In contrast, in insects and birds the bearing elements are made of dead cells and are therefore unrecoverable. Frayed wings are one of the most common symptoms of senescence in *Drosophila*. In insects, these elements are programmed as non-replaceable, whereas in birds they are replaced by new feathers during moulting. Hence, worn-out feathers are not a symptom of senescence because it is prevented by moulting. Another example is equally informative. Human beings possess two sets of teeth. The first juvenile set needs replacement due to the growth of the skull. When growth ceases and adolescence ends, the ability of replacing the adult teeth system disappears. These unrecoverable structures easily undergo deterioration, and their loss not only lowers the ability to properly disintegrate food, but also has dangerous life-threatening effects. Elimination of the program of teeth replacement has therefore far reaching consequences not only as a symptom of senescence (age of horses, for instance, is evaluated on the basis of the condition of their teeth), but also because it strongly influences the chances of survival of an individual. In contrast, incisors of rodents cannot be treated as a measure of age or senescence as these intensely exploited parts of the body grow continuously like claws or hair. Consequently, life or developmental programs determine whether the effects of unavoidable stochastic processes are visible (organism senescence) or whether the organism behaves as if young forever.

## Cellular- vs. organismal-level aging

Human body is built from approximately 10^13^ cells that form its dominant part in terms of mass. Being a multicellular organism means that all body cells must be integrated with the systems which enable its proper functioning. The system of body fluid circulation ensures immunity, cell nutrients supply, removal of waste products, transport of signal molecules and frequent oxygen supply. We have named this system “the internal environment” [5]. Homeostasis released somatic cells from the necessity to cope with problems related to constant changes in the surrounding. In this way, cells were able to specialise in various functions, in turn assuring proper functioning of the organism as a whole. It seems therefore logical that the question of the contribution of cells and internal environment to senescence and longevity is of high importance.

Until very recently, gerontological studies focused on cellular senescence and organismal**-**level senescence processes. Such a view of aging clearly suggests that certain mechanisms have evolved at the cellular level to participate in the organismal**-**level senescence and death. These mechanisms could not have evolved in unicellular organisms without a reason because for these life forms cell reproduction is equivalent to organismal reproduction, which in turn assures continuity of life. The existence of a mechanism equivalent to clonal senescence in unicellular organisms would be lethal to them. “Replicative aging” (i.e. the limit of the number of mitotic cycles of a single cell) of unicellular budding yeast is a side effect of the choice of a specific mechanism of cytokinesis [11] possible only in cells with rigid cell walls that are additionally strengthened with chitin rings. Such effects of evolutionary peculiarities are, however, negligible from the point of view of growth in the population of these species as the cells that stop reproduction are practically undetectable [16]. The opinion that the mechanism leading to unavoidable death of an organism has evolved as such is in clear conflict with the generally accepted rejection of Medawar's idea of purposeful aging [17]. The recent paradigm shift [18, 19] has revealed that “cellular senescence” is a misnomer for the process which is not functionally connected with programmed organismal senescence, but is rather a part of developmental biology. The process is involved in the replacement of unwanted cells already functioning since the embryonal stage of development. Hence, the paradigm shift additionally confirms the opinion of lack of “purposeful” nature of aging.

The process of “cellular senescence” may secondarily participate in deleterious processes in the elderly when the number of the “senescent” cells increases and contributes to local inflammation. Earlier understanding of the role of the process of cellular senescence led to using the unicellular budding yeast *Saccharomyces cerevisiae* as an important model organism in gerontology: *“Budding yeast is a preeminent model organism in studies of cellular aging pathways that are conserved in eukaryotes, including humans”* [20]. However, when the paradigm shift took place, the rationale for the use of a unicellular organism as a model for gerontological studies ceased to exist. Negative effects of “senescent” cell accumulation (inflammation) on multicellular organisms are a result of attracting killer cells after the end of cell division, not because cells are unable to proliferate further. According to the basic assumptions of yeast gerontology, yeast cells that ceased reproduction are considered dead. Consequently, the post-reproductive period of cell life does not exist, which in fact is not true [21, 22]. Effects of death of a yeast cell are, however, positive for its neighbours, not negative as in the case of animals, ensuring provision of nutrients for the survivors during the so called “chronological aging”. Hence, the conclusions drawn from gerontological studies on yeast are additionally internally inconsistent.

The only cellular**-**level phenomenon that has an impact on aging of higher-level organisms is absence of telomerase in some somatic cells. Indeed, restoration of telomerase activity leads to increased lifespan in mice [23]. However, one cannot rule out the possibility that absence of telomerase is a consequence of an accidental mutation that triggers late negative effects and cannot be eliminated by natural selection. Lack of increased frequency of neoplasia in the transformed mice seems to support this view because telomere shortening was postulated as the mechanism of preventing uncontrolled proliferation of cells. Consequently, even the mechanism leading to organismal senescence does not support the opinion that “purposeful” aging mechanisms can evolve in Metazoans. Again, unicellular yeast did not prove to be useful in explaining that trait because telomerase is active in the yeast cell throughout its life [24].

Even more damaging is the fact that the use of unicellular organisms to explain universal mechanisms of aging was based on erroneous premises. Uncritical extrapolation of the conclusions drawn from the behaviour of parts to the whole system, known as *pars pro toto*, is regarded as one of the basic errors in reasoning. However, the most fundamental error of yeast “replicative aging”, which can be now regarded as a biological curiosity, was the use of the number of daughter cells produced by a single cell as a measure of age and longevity, rather than units of time. It has been recently revealed that these units are neither the same nor proportional, which makes any rational discussion next to impossible [22, 25]. It is hardly surprising therefore that yeast “longevity mutants” appeared to live as long as the standard strains and even the “short lived” mutants [26].

Hence, although cells are a dominant part – in quantitative terms – of a multicellular organism, the process of human or animal aging mostly comprises negative consequences which affect the organismal-level processes. It can be argued that the death of a few cells specialised in production of signalling molecules can strongly influence human aging. The problem with that argument, however, is that the death of such cells is triggered by organismal-level processes and does not result from intrinsic mechanisms limiting cell reproduction. Furthermore, negative consequences of the activity of epigenetic maintenance systems applies most likely to those cellular processes that regulate organismal rather than cellular-level phenomena. Therefore, the postulated weakening of cell housekeeping functions by accumulation of various ballast molecules does not seem to be valid from the perspective of human aging [5]. Paradoxically enough, accumulation of lipofuscins in post-mitotic cells is considered to be one of the most important “senescence markers” [27], although there is no proof that such cellular-level waste retention measurably influences human longevity or senescence, except for some “cosmetic” effects. Evolution of cellular protective, repair and turnover system had enough time since the Precambrian to prevent most of the negative effects of the passing of time.

One of the examples of misinterpretation of the known facts is the discussion concerning the universality of sugar sensing in unicellular and multicellular organisms. The fundamental difference between these two types of organisms is that unicellular life forms have to monitor changes in the environment to adapt their behaviour to the expected drop in the sugar level in order to avoid starvation in the event of sugar depletion during the cell cycle. Unicellular organisms are unable to secure for themselves a continuous availability of sugar outside the cell. In contrast, the majority of human somatic cells live under the conditions of homeostasis ensured by a perfectly regulated system of internal environment [5]. Insulin-like proteins could only have evolved in multicellular organisms. In these organisms, a few specialised cells producing insulin and glucagon play a regulatory role in ensuring the continuous level of glucose in internal environment. An example of an executing organ is the liver. Hence, the majority of somatic cells have no problem with sensing or responding to changes in the environment. In fact, similarity of sugar-related proteins engaged in both uni- and multicellular organisms has its roots in the origin of protein structures adapted for sugar binding, not the functions played by them.

## Instances of aging in animal world

Experimental gerontology is based on the assumption that the phenomenon of aging is of a universal nature and that the mechanisms of aging have been conserved and are the same in all organisms. This has led to the extrapolation of conclusions about human aging drawn from studies conducted on organisms that are evolutionarily and structurally distant from humans. Some of the most recent opinions are worth citing *expressis verbis*: *“Finally, we believe there is good evidence for universal mechanisms of aging (at least between fungi and metazoans). Parsimony suggests that the ubiquity of aging is likely the result of conserved mechanisms of aging. (…) We believe that yeast is a valuable model system for aging that will continue to contribute to our understanding of aging generally”* [28]. Some consider aging a universal trait [29]. Others claim that aging prevails at least in animals [3]. Only one of comparatively recent papers [30] mentions that aging is not universal among animals.

We conducted a survey of the incidence of senescence phenotype among various animal clades. Only in a few groups was a certain uniformity found. In most groups, we found high diversity in the length of life as well as the appearance of senescence symptoms. This diversity made it difficult to draw any reasonable conclusions. We assumed that we will assign a particular trait to a group, if it clearly occurs in at least one of subgroups. That assumption results from the following reasoning: features such as constant body growth, agametic reproduction or regeneration ability can hardly be acquired independently *de novo* by only a small subgroup of organisms as they need a perfectly coordinated expression of very complex sets of genes. On the other hand, disappearance of such traits in another subgroup might result from mutations only in a very limited number of regulatory genes.

At the end of this article we will propose an explanation of what mechanisms are involved in the emergence of short-lived phenotypes among species representing groups of animals with potentially high longevity phenotypes while not showing symptoms of senescence. However, one rule has already become clear: short-lived and senescent phenotypes are observed in species of a small body size.

The simplified tree of life of animals presented below shows that aging prevails mainly in life forms that human beings encounter in their close surroundings. One can easily spot not only aged mammals, birds and insects, but even aging trees and mushrooms. This clearly suggests that the opinion of ubiquity or universality of aging is a simple consequence of superficial observation focused only on terrestrial life forms. On the other hand, in aquatic environments, unlike the terrestrial ones, biologically immortal and non-senescent forms prevail. We have to bear in mind that life on land appeared much later than in water. Terrestrial animal forms evolved from different aquatic clades and therefore cannot have a close common origin. Taking into account how evolutionarily distant insects (*Drosophila*) or roundworms (*Caenorhabditis*) are, and how different their anatomy and biology is when compared to mammals, one can hardly expect them to have common mechanisms of aging. Rare occurrence of senescent forms in aquatic environments suggests therefore that claims concerning the conserved character of aging, its ubiquity and universality are a clear and misleading oversimplification.

## Which living objects age?

An important question should be asked, namely which living objects age. One of the rare papers accepting lack of universality of aging among animals [30] presents an opinion that claims attributing the existence of aging to sex are false. We believe that the rejection of the above generalization is only formally right.

Senescence and unavoidability of death can be attributed to terminal differentiation in sexual reproduction, not sex itself. For instance, the agametically reproducing immortal *Hydra* also reproduces sexually, but is not terminally differentiated to do it [31]. Terminal differentiation, however, can be found in another cnidarians, such as jellyfish (stage of medusa). Hence, all life forms irreversibly specialised in sexual reproduction are generally mortal and often senescent. Cases such as parthenogenesis do not invalidate the rule. These organisms produce progeny using all the machinery necessary for sexual reproduction and no cell of their body (soma) can be found in the progeny. Life of these individuals (clones) starts from a single cell of the germline and hence the basic part of the sexual program is fulfilled in them. These organisms meet the criterion of being the soma. In contrast, agametically reproducing animals are biologically immortal, but in their case the body of the initial organism directly participates in the formation of the progeny.

## Biological immortality and mortality without concomitant symptoms of senescence

Development of senescent phenotypes is clearly a late-appearing trait among animals. It involves only the last steps of animal development during which the organism is able to reproduce exclusively sexually. This is best illustrated by the emergence of senescence even in the simplest animal forms, like cnidarians, where we encounter immortal forms like hydra, which, besides being capable of agametic reproduction, can also reproduce sexually. Such type of reproduction is realised in the form of budding of the polyp at the same life stage. Sexual reproduction of these animals does not require any specialisation. In other organisms from the same group (jellyfish), their earlier forms (polyps) reproduce agametically by strobilation. They can, however, enter the second step of development, namely the formation of the medusa, in which they can only reproduce sexually. In most species of these cnidarians, medusas start to show symptoms of senescence and die after sexual reproduction. Yet in at least one species, *Turritopsis dohrini*, this step can be reversed to the stage of the polyp, which again can reproduce agametically and become immortal [32]. This is so far the only known case when senescence does not lead to unavoidable death.

One possible generalisation seems to be allowed. Mortal forms exist when an organism is highly specialised in reproduction, irrespective of its mechanism. Agametic reproduction *sensu stricto* applies mainly to sessile life forms. Dissemination of species to far distances can be achieved by the formation of motile life forms dispersing actively, or by wind or water currents. Consequently, larvae are motile or float passively in water environments, which ensure dispersion of the species and population of new habitats. This is true not only in the case of animals, and not restricted to sexual reproduction. Mortal conidiophores of moulds also disseminate long distances by wind while their sexual forms have lower chances of spreading. Sexual forms of basidiomycetes, like fruiting bodies or stalks of slime moulds, are also mortal, and serve only as a means of dispersing species significantly farther than can be done by the “immortal” mycelia or single cell forms of slime moulds living in soil.

All these non-animal forms of life, including trees or seed sprouts of other plants, have all features ascribed to the soma of mortal animals. All of them have also one additional feature in common. They are individuals, which means they are recognisable from the moment of birth or formation until their death by the spatial location and acquired features. Hence, being the soma means being an individual.

## When is senescence encountered in animals?

Symptoms of senescence manifest only when allowed by the selected life program. More precisely, senescence takes place when the program of cell replacement and regeneration is turned off or irreversibly lost. For example, the program responsible for cell replacement/regeneration is turned off during the transformation of insects into the sexually competent imago stage. With a few exceptions, soma of imagoes is built from post-mitotic cells, which precludes replacement of any dead or non-functional cells. In mammals, at the end of adolescence cell replacement mechanisms are restricted only to some types of cells; during evolution of this particular group, the enormous regenerative capacity of our early progenitors was irreversibly lost, except with regard to the liver and the constant growth of hair or nails, but surprisingly not the equally important teeth. Consequently, senescence in mammals has a completely different scale when com-pared to adult forms of insects, roundworms or rotatoria.

In the other words, constant ability of replacing worn out cells and some organismal-level structures prevents appearance of symptoms of senescence in continuously growing species, like representatives of crustaceans, molluscs, fish, amphibians and reptiles. As a result, cellular-level aging cannot play a crucial role in their unavoidable death as aged cells are replaced whenever possible.

Summary of these considerations is presented in Figure 2.

**Figure 1:**
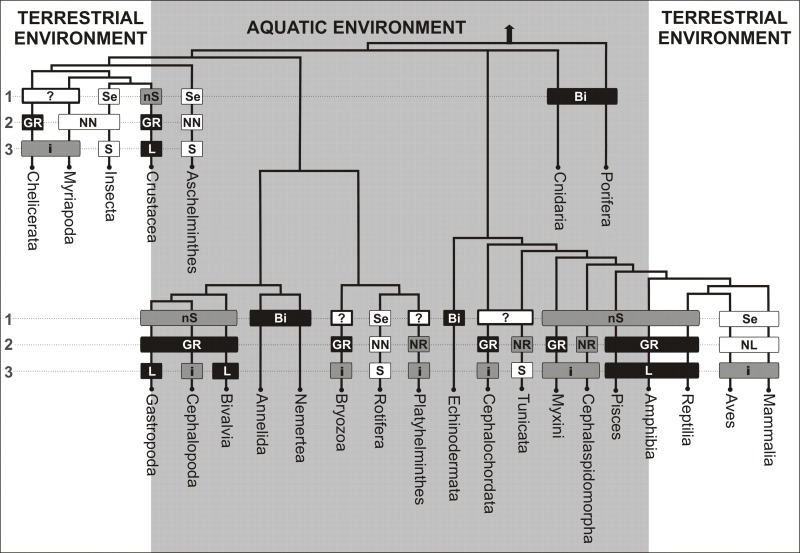
The senescence phenotype types among various animal clades Symbols and abbreviations: **1 - Type of senescence** (**Bi** - biologically immortal, **nS** - mortal: nonsenescent, **Se** - mortal: senescent); **2 - Growth** (body size increase of adults) and regenerative abilities (**GR** - constant growth and high regeneration, **NR** - no growth and high regeneration, **NL** - no growth and low regeneration, **NN** - no growth and no regeneration); **3 - Lifespan** (**L** - long, **i** - intermediate, **S** - short); Pisces - Classes: *Chondrichthyes, Sarcopterygii and Actinopterygii*.

**Figure 2:**
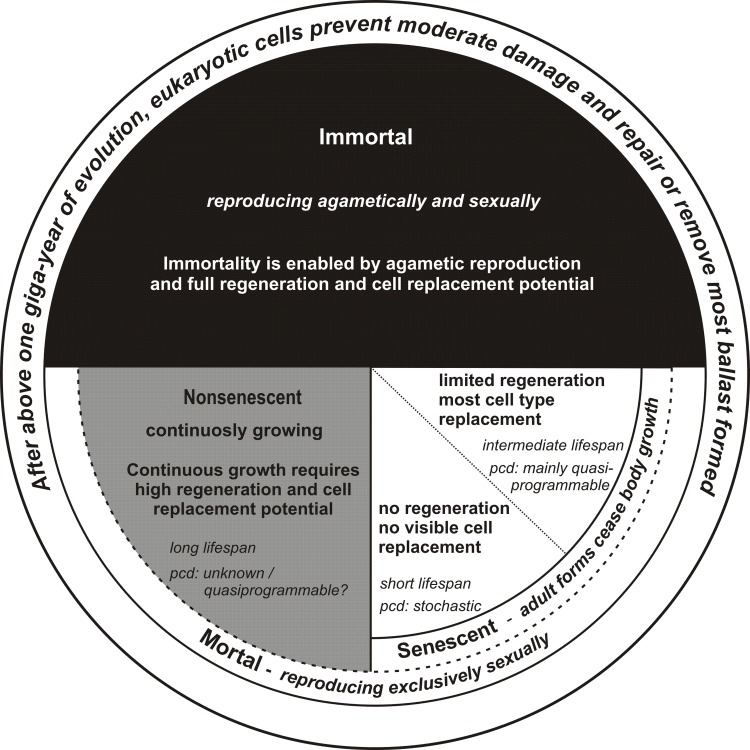
The relationship between senescence and life program connected with the ability to cell replacement and regeneration pcd – proximal cause of death.

## Two main types of senescence

The prevalent opinion that organismal senescence is strictly connected with lower efficiency of cells resulting from accumulation by cells of various toxic or ballast structures generated by stochastic processes finds no convincing experimental support [5, 33]. Strictly speaking, there is little evidence of the effects of such accumulation on organismal senescence. Cells have evolved over giga-years, and are extremely well-equipped to prevent such negative effects. Mechanisms of preventing such damage are ubiquitous and qualitatively as well as quantitatively comparable, irrespective of the taxonomic group. Similarly, mechanisms of repair and replacement of worn-out molecules and cellular structures are omnipresent in eukaryotic cells. In contrast, organismal-level mechanisms emerged more recently, and continue to evolve under the pressure of the changing environment. Senescent cells show a specific “senescence phenotype” characterized by morphological and physiological alterations including changes in the expression of genes related to important metabolic processes [34]. Negative effects of “senescent” cell accumulation on multicellular organisms are a result of secondary effects (*senescence-associated secretory phenotype*, SASP phenotype) appearing in already non-dividing cells, not because cells are unable to proliferate further. The basis for this phenomenon is the increased secretion of a number of environmental factors such as – pro-inflammatory cytokines and chemokines, growth factors, proteases, or ECM (*extracellular matrix*) components (i.e. fibronectin) [35]. The influence of senescent cells on the functional efficiency of tissues seems to be clear; however, the causal role of cellular senescence in age-related degeneration does not seem so clear and remains a speculation [36]. However, recent findings suggest that selective elimination of senescent cells leads to extending the lifespan of mice and can ameliorate some age-related disease processes [37].

All living things are subject to various destructive forces, extrinsic as well as intrinsic. Damage to cells and organismal-level structures is a constant process. These forces can kill the organism, if their intensity exceeds the organism's recovery capacity. The survivors are either able or unable to replace damaged or worn out parts or cells; in the latter case, they show symptoms of senescence. This inability is primarily a consequence of the selected life program, rather than an effect of the operation of the Second Law of Thermodynamics.

The title of the paper by Leonard Hayflick that has strongly influenced our thinking starts with the following postulate: “*Entropy Explains Aging, Genetic Determinism Explains Longevity, and Undefined Terminology Explains Misunderstanding Both”* [10]. This statement clearly suggests the existence of entirely different mechanisms in the two components of the term aging, which are inseparable in human beings. The first part suggests a clearly stochastic nature of the senescence process, whereas the second part points to the genetically encoded longevity of species. Below, we postulate that senescence is driven by stochastic processes only in certain groups of animals represented by insects, roundworms, or mortal forms of immortal species such as medusas. In the case of mammals and birds, quasi-programmable mechanisms of senescence prevail, despite some rare exceptions.

### Senescence resulting primarily from the stochastic mechanisms of wear and tear (insects, roundworms and rotatoria)

The result of an almost complete lack of replacement/regeneration capacity in adult forms of insects is that imagoes cannot neutralise effects of various kinds of accidental or stochastic wear and tear processes affecting the organismal level. This eventually leads to death of the individual. The fate of bee workers is the best illustration of that problem. The bees that have broken their wings when collecting nectar or pollen die of hunger in the field. Those with worn out, frayed wings can be useful within the hive as long as, say, their joints allow it. Wear and tear processes impacting a part of the body as important as wings support the view that death of cells is not necessarily connected with the cell death resulting from damage. Most of the wing is built from cells that are already dead at the time they are formed. Further, one can imagine other proximal causes of death of wear and tear processes, which we will describe as “breaking the weakest link in the life chain”. For instance, among bees, the queen is much less physically active and lives longer than other individuals, especially since it is under the special care of the community. Its death results probably from breaking different weakest links. Hence, accidental and stochastic processes dominate as causes of appearance of symptoms of senescence and are responsible for the short life of adult forms of insects. Furthermore, that short life results from lack of cell replacement systems (the soma consists of post-mitotic cells) and also lack of the inducible immune system present in vertebrates.

### Quasi-programmed senescence

However, things are quite different in mammals. M.V. Blagosklonny claim that aging of mammals has a rather quasi-programmed character (i.e., is a spandrel): “*Recent discoveries suggest that aging is neither driven by accumulation of molecular damage of any cause, nor by random damage of any kind”* [38, 39]. Mechanisms involved in senescence of mammals are presumably not uniform. Quasi-programmed aging is defined as a non-adaptive continuation of developmental processes during adulthood. The result of this is hyperfunction thus accelerating the development of various age-related pathologies and diseases. The hyperfunction theory provides an alternative explanation to molecular damage as a central mechanism of ageing [38-42]. One group of such mechanisms results from hyper-functioning of various organismal-level processes when body size increase ceases at the end of adolescence. These processes can be partly slowed down by preventing energy excess [4]. Another mechanism may include hyper-function of epigenetic maintenance systems which turns off important functions of cells. Methylation of promoters of genes that are crucial for cell functioning or of genes producing important structural (e.g. collagen) or regulatory proteins necessary for higher level functions of organisms leads to the slowing down of life functions of the organism as a whole. The mechanisms of DNA methylation, if hyperactive, can turn off necessary genes on one or both copies in some cells, giving rise to stepwise loss of various functions of the organism as a whole. A low level of DNA methylation in centenarians strongly supports such an explanation of programmable origin of human senescence [43]. Organisms that grow continuously, such as big reptiles or crustaceans, resemble adolescent humans and most likely are capable of eliminating these dysfunctional cells or replacing the dead ones, thus preventing the symptoms of senescence. High level of heterozygosity of human populations can explain why various populations and individuals show different rates of senescence and longevity.

A limited role of external stochastic factors on human longevity cannot be ruled out, as for example when contact with particular foreign antigens can induce autoimmune aggressive response or as in the case of decay of the teeth system.

## Environmental factors determining short lifespan of representatives of potentially long lived groups of species

Aging, understood as a progressive decrease of life functions (senescence) and increased mortality, is also observed within groups of organisms encompassing numerous long-lived species manifesting no visible symptoms of senescence. We consider these phenotypes as resulting from secondary processes spurred by environmental factors. It is necessary to bear in mind that in the presented tree of life, a specific aging phenotype was assigned to a particular taxonomic group, if at least some representatives within the group presented obvious high longevity accompanied by lack of organismal senescence symptoms or biological immortality. We presumed that lack of the longevity phenotype, especially in small short-lived species, could have a secondary origin. A possible mechanism of shortening lifespan of some species will be discussed below.

### Extrinsic mortality

One of the most important factors influencing longevity of species is extrinsic mortality, which results mainly from predation. The higher the pressure from predators, the shorter lifespan is observed. However, very short-lived phenotypes are also observed in nutrient-rich ephemeral aquatic environments eliminating adult individuals within one season. Similarly, high availability of food exists on land, primarily close to and just below the soil surface. Life in burrows assures availability of green parts of plants as well as roots and tubers. As a consequence, these places are inhabited by a large number of small animal species. However, the pressure of predators is also very high there, leading to a shortened lifespan among plant-eating animals. Naked mole rats are an exception to this rule because they create a deep underground burrow system partly sealed from the external world. Their eusociality and presence of soldiers further limits predation, allowing for increased lifespan [44]. Low pressure from predators is similarly observed in numerous inhospitable environments, such as deep sea and saline or freshwater caves, where low density of pray populations prevents or substantially lowers the presence of predators. Hence, low extrinsic mortality promotes longer life [45].

Living in environments driving frequent generation turnover has an obvious consequence. Animals living under such conditions cannot be big because growth and maturation take time. In addition, life in burrows promotes small size for purely mechanistic reasons. Similarly, mechanistic considerations prevent birds from continuing to increase in size throughout their life – constant growth would exclude particular types of flight for purely physical reasons. Consequently, by choosing these types of environments, representatives of groups of potentially long-lived animals, such as reptiles, increased their rate of reaching sexual maturity under the pressure of external high mortality. Genes assuring longevity (including those conditioning intense constant growth) and preventing appearance of the senescence symptoms were able to mutate, as their expression appears in the “shadow of natural selection”.

### Food availability

Availability of food or energy source is generally a prerequisite for continuity of life. In most environments, the availability of food undergoes seasonal changes. It is well known that in certain animals, such as for instance echinoderms or annelids, excess of food induces agametic reproduction both in larval and adult forms. When food is easily available, this sometimes takes a form of body fission. This, by far, is a quicker way to increase the number of sexually mature individuals compared to the time-consuming sexual reproduction. The mechanisms assuring constant availability of energy necessary to survive periods of scarcity evolved both at the cellular and organismal levels. It was already postulated [4, 46, 47] that in organisms such as mammals and birds, which stop growing after reaching sexual maturity, excess of energy can have a strong negative effect on the length of life of individuals. In contrast, such a problem does not exist in organisms that can invest excess food in any kind of reproduction or utilize it to increase their body size. In fish the number of rings corresponds to the number of years lived, but their width describes annual availability of food. During the periods of plenty, excess of energy is directed to the body growth. As such, the role of constant body growth in preventing negative consequences of excess food availability is clearly visible. However, high longevity of continuously growing animals results mainly from constant replacement/regeneration systems and only to some extent from preventing negative consequences of energy excess.

The constantly low rate of resources renewal encountered in some environments, such as freshwater or saline caves, deep see communities, and soil or timber, promotes longevity phenotypes. Such phenotypes can, however, be expressed only in the case of strongly lowered extrinsic mortality. Further, when analysing biology of various groups of mammals, it becomes clear that their inherited possibilities of growth, cell replacement and regeneration are not identical. For example, huge arctic whales live very long, showing an ability to increase their size to the degree impossible on land due to mechanical reasons. On the other hand, generally short-lived rodents have incisors growing throughout their lives. Some rodents can easily regenerate lost skin when caught by predators. However, these specific features of rodents do not increase their lifespan but merely increase their chances of survival.

## Relations between senescence and age-associated diseases

Naked mole rats (NMRs) represent mammals showing extraordinary longevity when compared to their close relatives of the same body size and instead in line with the longevity of the biggest representatives of the rodent group, like capybara. In addition to unusually high longevity, these species show no signs of senescence almost until the end of their long lives [48]. This pattern of senescence has been termed “delayed senescence” to distinguish it from the negligible senescence of long-lived reptile and fish species. The mechanism of evolving a phenotype which is so unusual among mammals has not yet been identified, albeit it was postulated in our previous paper [4]. Human centenarians show clear symptoms of senescence in contrast to NMRs, which additionally reproduce until the end of their lives. From this, one can draw a conclusion that the reproductory system of NMRs does not senesce. Increased longevity enables survival of the species under the conditions where food scarcity evidently restricts the size of litter to one per season, in contrast to the notorious fecundity of their short-lived rodent counterparts. This severe food limitation, accompanied by simultaneous elimination of age-associated diseases and senescence, allows for production of a sufficient number of progeny to counterbalance unavoidable natural mortality. In human gerontology, it was previously observed that after reaching a certain advanced age the number of typical age-associated diseases falls. Centenarians are only devoid of those inheritable factors that eliminate age-associated diseases, not the typical symptoms of senescence. Centenarians represent only a small fraction of human population due to high heterozygosity of the population, in contrast to NMRs.

As such, it is difficult to firmly establish causal relations between senescence, age-associated diseases and the maximum lifespan. Relationships between senescence and age-associated diseases are especially intriguing. The possibility of eliminating causes of most age-associated diseases in humans and NMRs and simultaneous presence of typical senescence symptoms even in long-lived humans strongly suggests that both phenomena are encoded in a different way. Some overlapping between these processes is, however, possible. For example, senescence of immunological system can secondarily influence the mortality rate in the case of age-associated diseases [49]. The situation observed in humans can be described in the following way: if an individual did not inherit susceptibility to most of the age-associated killing diseases, it would eventually die because of senescence of one of the most worn-out body elements. In other words, the rate of senescence may determine the maximum lifespan of senescing animal species. The already quoted opinion of Leonard Hayflick, who suggests different origin of both processes, is worth remembering here.

## Problem of causes of death of long-lived animals showing no symptoms of senescence

The problem arises when we try to answer the following question: what could be the proximal causes of death of the negligibly senescent (continuously growing) organisms? Some of them will die because of purely accidental reasons. Others will die because of what we propose to name “breaking of the weakest link in the life chain”. No biological program provides solely life-supporting features. In fact, inherent negative consequences can be found in any program. Continuous growth of the cell or the body has obvious handicaps. For example, a continuous growth of dinosaurs living on land was limited by purely mechanical reasons, such as strength of their bones and muscles, efficiency of the circulatory system, and even temperature regulation. The efficiency of the body cooling mechanism can be limited above certain level of heat production increasing proportionally to the body mass. Similarly, cell volume cannot increase infinitely because of the surface-to-volume relation (uptake) and cellular communication by diffusion. These factors can limit the maximum lifespan, although no symptoms of senescence are visible in these species.

## General conclusions

In human beings the term “aging” means appearance of symptoms of senescence and increased probability of death at advancing age. However, after an analysis of various life forms, one can conclude that senescence and unavoidability of death in general are at least partly separable in mortal organisms, while numerous groups of simpler animals are biologically immortal. The phenomenon of senescence is observed in those species or life stages of organisms that cannot by principle remove the damage done by various adverse extrinsic and intrinsic forces. An analysis of differences in life programs among various taxonomic groups of animals as well as within a particular group allows for a generalisation that there are three main aging phenotypes. The first encompasses representatives of the simplest animals like sponges, cnidarians, annelids, nemerteans or echinoderms that show biological immortality, that is, lack of intrinsic causes of death. These animals rarely manifest symptoms of senescence. The reason for their immortality is the ability to reproduce agametically (besides sexually), resulting from the enormous ability of cell replacement and regeneration. The second group is represented by the organisms which, while being mortal, show no visible symptoms of senescence. This phenotype is a consequence of the constant increase in body size after reaching sexual maturity. Because proportional growth requires constant availability of most of organismal-level developmental programs, such constant growth is accompanied by high cell replacement and regeneration ability. The best known representatives of that group are crustaceans and molluscs among invertebrates and fish and reptiles among vertebrates. Constant growth corresponds to the adolescence period of mammals or larval stages of insects as these animals do not show organismal-level senescence. Consequently, continuously growing animals are “young forever”.

The third and very diverse group is represented by insects and roundworms among invertebrates and mammals and birds among vertebrates. These animals show evident symptoms of senescence but differ in longevity. Their adult representatives live for a very short time. The presence of symptoms of senescence in these animals results from their primary life programs. Their senescence is a consequence of the lack of, or very limited, cell replacement and regeneration mechanisms of imago.

However, within those groups which we describe as non-senescent, there exist short-lived taxons manifesting the senescent phenotype. We postulate that these taxons evolved as a consequence of secondary processes in response to the demands of specific environments. As a rule, these species are small animals reaching sexual maturity within a short period of time. This phenotype evolved, for instance, in response to the conditions of ephemeric aquatic environments or as a result of high pressure from predators.

Unavoidability of death affects those life forms that are terminally differentiated in sexual reproduction or, more generally, the type of reproduction assuring easier dissemination (dispersal) of species compared to agametic reproduction.

The practical conclusion that can be drawn from these considerations is that lack of universality of aging suggests a fundamental change in approach to gerontological problems. Instead of looking for mutants of simple and evolutionarily distant species with increased lifespans, gerontology should focus on finding factors alleviating the most life-disrupting effects of senescence.

## Glossary

Aging: The term refers to accumulation of negative consequences of passing of time (senescence) and increased probability of death. Due to its ambiguity, this term should not be used in scientific publications, except when describing this dual meaning.
Cellular and organismal-level waste retention: These processes of various origins can be either neutral or potentially harmful. The most harmful are those concerning the organismal level, e.g. atherosclerotic plaques, gall or nephric stones. At the cellular level, retention depends on the rate of their formation and dilution during cell division. The accumulation of lipofuscins in post-mitotic cells results simply from their resistance to enzymatic degradation and inability of dilution during subsequent mitotic cycles.
Internal environment: Comprises extracellular space of a multicellular organism under control of the organism as a whole. The term applies mainly to the space to which body fluids have open access.
Senescence: Describes complex negative changes observed in organisms during the passing of time.
Spandrel: A trait which is not genuine and evolved as a side effect of an original trait.
Weakest link in the life program: A trait which is a built-in side effect of the chosen life program, causing death of an organism which shows no symptoms of senescence.
Wear and tear processes: This term refers to organismal-level effects of operation of stochastic or purely accidental destructive processes. The presence of such processes could be transient and cannot be identified with senescence.

## Abbreviations

NMRs: naked mole rats
SASP phenotype: senescence-associated secretory phenotype
